# Impact of COVID-19 on Management, Quality and Satisfaction of Health Organizations: A Case Study in a Portuguese Hospital

**DOI:** 10.1007/s41742-022-00505-7

**Published:** 2023-01-27

**Authors:** Tânia Gaspar, Vanesa Salado, Maria do Céu Machado, Fábio Botelho Guedes, Manuela Faia-Correia, Anabela Coelho

**Affiliations:** 1 Universidade Lusófona das Humanidades e Tecnologias/HEI-LAB, Campo Grande 376, 1749-024 Lisbon, Portugal; 2 Portuguese Lab for Healthy Workplaces, Lisbon, Portugal; 3 ISAMB/Lisbon University, Lisbon, Portugal; 4 Sevilla University, Seville, Spain; 5 COMEGI-Centro de Investigação em Organizações, Mercados e Gestão Industrial, Lisbon, Portugal; 6 Universidade de Évora, Évora, Portugal; 7 Comprehensive Health Research Centre (CHRC)/Évora University, Évora, Portugal

**Keywords:** COVID-19, Health organizations, Quality of health, Satisfaction of health professionals

## Abstract

The hospital health care professionals are the front-line fighting COVID-19 considering they are responsible for all the care provided to patients. The purpose of this study was to determine the impact of COVID-19 at the hospital management level and, also, to understand how psychosocial environment, and satisfaction of Health Professionals were affected. A case study was performed in a Portuguese Hospital. Data were collected at one hospital under study at two different occasions: the first before the pandemic (November 2019) and the second almost two years after the pandemic started (November 2021). Regarding data collection, 37.0% of participants responded in the occasion 1 (*n* = 296) and 63.0% responded in the occasion 2 (*n* = 503). The instrument for the Assessment of Global Management of Health Organizations (AGMHO) consists of 39 items organized into six dimensions (Gaspar et al. in J Occup Environ Med 63: 581–587, 2021). Comparing timings pre and during pandemic COVID-19, it was found that the participants in the pre-COVID-19 era showed stronger organizational culture, higher quality of life, better psychosocial environment regarding content/leadership and higher job satisfaction when compared to the participants during pandemic COVID-19. On the other hand, participants in the second occasion were found to have higher psychosocial risks related to mental health when compared to participants in the pre-COVID-19 phase. We conclude that the professionals’ perception of the different dimensions of the health organization worsened after 2 years of the pandemic. With special focus on psychosocial risks at work and relationship with leadership.

## Article Highlights

- The systemic evaluation of professionals regarding the quality of the health organization worsened 2 years after the start of the pandemic.
- After two years of facing the pandemic, professionals were exposed to high levels of stress and higher psychosocial risks at work.
- The professionals' satisfaction reveals that it is more strongly influenced by the psychosocial environment related to the work content and leadership relationship, as well as the organisational culture.
- It is a priority to intervene in leadership relationships and psychosocial risks at work related to the physical and psychological demands of work, stress management and a more active and involved role for the professional.
- The importance and impact of organizational culture on processes and results was demonstrated.
- The relationship between QoL and psychosocial risks of work with other processes and outcomes of health care organizations was confirmed.
- A regular evaluation of health organizations is proposed, using systemic models that allow evaluating, monitoring and supporting evidence-based governance process.

## Introductions

The history of humanity reminds us of several set of epidemics and pandemics, like cholera, plague, and others, which definitely changed the course of public health, human behavior, social relations and economic environment.

The actual COVID-19 global crisis, announced in Wuhan City in China, since December 2019, proved how daily lives can be changed, and people forced to move on through their work and social relations in a totally different approach.

According to the latest data of World Health Organization more than 433 million confirmed cases and over 5.9 million deaths have been reported globally (WHO 2022).

The European Centre for Disease Prevention and Control (2020) highlights the increased risk of infection in health care professional considering their daily life work with patients and population in general and their enormous risk to emotional distress (Hajure et al. 2020) in line with the worldwide phenomena of the increasing prevalence of anxiety and depression, in general population, and the highest rates of mental distress correlated with periods of confinement and intensifying of COVID-19 deaths (OECD 2021).

Several studies highlight the psychological damage done upon the health care professional that were infected with COVID 19 and upon their families due to the fear of transition of the disease, death, being quarantined etc. (Mohammadian Khonsari 2021; Pfefferbaum 2020; Zandifar 2020).

In Portugal, from March 2020 to January 2022, around 2 566 551 patients were diagnosed with COVID-19 and 53% were women (31% of which had more than 40 years old) (DGS 2022). It was, also, estimated that more than 22,000 Portuguese health care workers have been infected by COVID-19, in their professional context (Campos 2021).

Data from DGAEP in September 2021 shows that more than 96. 771 health care workers were allocated to the Portuguese health care institutions: 53.451 nurses, 32. 409 doctors and 10. 911 diagnostic and therapeutic technicians (Directorate-General for Administration and Public Employment 2021).

Most of the health care force is supported, globally, by women, data from WHO reports that 70% of the health care workforce are women, and they are still lower paid, with lower status facing harsh realities of gender bias and harassment (Gaspar et al. 2021; WHO 2021). The COVID-19 crises exacerbated the situation and recent evidence, on the impact of COVID-19, suggests that women’s economic and productive lives were more affected than men. More women have jobs that expose them to infection and psychological stress and with the widespread closure of schools and childcare facilities women were forced to, additionally, supervise or lead home-schooling (OECD 2020).

The permanent gender inequalities across many dimensions expose women to the anticipated widespread economic fallout from the COVID-19 crisis and will amplify women’s unpaid work burdens (OECD 2021).

This whole predictable scenario should have triggered measures to protect and prevent occupational hazards, like exposure to SARS-CoV-2 and other pathogens, heavy workload, skin disorders and heat stress from prolonged use of prolonged use of personal protective equipment, psychological distress, chronic fatigue, etc. (WHO 2021), however, politicians and managers were not aware of this potential chaos.

It was also expected that healthcare professionals would experience mental health problems and work-related stress, during COVID-19 pandemic, which can lead to less satisfaction at work and decreased health and quality of life in the long period (Gaspar et al. 2021; Iskandarsyah et al. 2021).

If the mental burden of the disease may overwhelm the general population the impact on the health care professionals is much higher due to lack of human resources, excessive workload and permanent emotional management of patients and families in this context of a health crisis. Several studies show that health care professionals as the front line of care have high risk for developing stress, anxiety and depression, due to their role in management of patients (Mohammadian 2021).

To mitigate these hazards, a well-coordinated and comprehensive leadership that protects the health, safety, and wellbeing of health care workers should have been implemented. Measures for infection prevention and control, occupational health and safety, health workforce management and mental health and psychosocial support can reduce rates of work-related illness among health workers, absenteeism and less productivity (Van Gool et al. 2022; WHO 2021).

Some proposals have been endorsed to governments to guarantee adequate psychological counseling, a balanced work schedule with periods of rest and leisure for health teams during this pandemic crisis (Mohammadian 2021; Schmidt et al. 2022).

All the psychological consequences that health professionals are exposed increase burnout and consequently the degradation of the health system, with a non-expected human resources reduction, due to absenteeism and sick leaves (Rodríguez 2020).

Considering that leadership impacts the sustainable organizational environment through productivity, work satisfaction, work engagement, and work efficiency, managers should find high commitment together with health care professionals. Recent studies show that being in full pressure situations, health care professionals will not hesitate to give their best performance if well committed (Yáñez-Araque et al. 2021).

The main objective of this study is to understand and characterize the impact of COVID-19 at the hospital management level, psychosocial environment, and satisfaction of Health Professionals through the case study of a Portuguese public hospital before and almost two years after COVID-19.

## Methods

### Participants

Data were collected at one public hospital at two different moments in time: the first before the pandemic (November 2019) and the second two years later (November 2021).

With regard to the sociodemographic characteristics of the sample participated 396 health professionals, 247 female (83.4%). The majority (59.5%) reported being married, 20.3% single and 8.7% separated or divorced. Participants were aged between 23 and 68 years (mean 44.32, standard deviation 9.71, mode 40 and median 53). Regarding the level of education of the participants, it was observed that 12.8% had up to 12 years of schooling, 87.2% had a degree, Master’s or Doctorate.

With regard to the sociodemographic characteristics of the sample, it was found that of the 503 participants (health professionals), 82.9% were female (*n* = 471). Participants were aged between 21 and 65 years (mean 42.68, standard deviation 9.82, mode 42 and median 44), with 26.2% being single (*n* = 132), 64.0% married or living together (*n* = 322) and 9.3% divorced or separated (*n* = 47). Regarding the level of education of the participants, it was observed that 25.3% had up to compulsory education (12 years of schooling), 74.7% had a degree, Master’s or Doctorate.

In the second moment an open question was included to assess suggestions for improving health organizations, to which 192 participants responded.

### Instruments

The instrument included sociodemographic questions and six scales to evaluate the variables under study: organizational culture, quality of life, environment and psychosocial risks of work, performance management and professionals’ satisfaction with work.

#### Assessment of the Global Management of Health Organizations (AGMHO)

The instrument for the Assessment of Global Management of Health Organizations (AGMHO) consists of 39 items organized into 6 dimensions: The Organizational Culture dimension consists of 8 (*α* = 0.91), the Quality of Life by 5 items (*α* = 0.83), Psychosocial Work Environment related to work content and relationships with leadership has 11 items (*α* = 0.93), Psychosocial Risks at Work related to Well-being and Mental Health have 4 items (*α* = 0.83), Performance Management has 5 items (*α* = 0.91) and Professional Satisfaction has 6 items (*α* = 0.86). All questions have a 5-point Likert-type scale (Gaspar 2020; Gaspar et al. 2021). The questionnaire included an open question related suggestions for improving health organizations.

### Procedure

The data collection procedure included different phases. In the first phase, the study was submitted to the Ethics Committee of the Lisbon Academic Medicine Centre of the Lisbon North Lisbon Hospital Centre of the Faculty of Medicine of the University of Lisbon and obtained a favorable opinion.

For the implementation of the research study, and after the identification of the hospital that would be the target of the study and the approval of the respective administration, meetings were held with the clinical director and other manager professionals for the presentation of the project and involvement in the data collection process (Gaspar, Correia, and Torres 2021). After the project was presented to the administration and collaborators of the hospital and their agreement to participate in the study, it was further submitted to the Ethics Committees and Boards of Directors of the hospital.

After the obtained of all the necessary authorizations, the data collection was started. The quantitative instrument was applied through a link which was disclosed to the participants.

For any of the instruments, anonymity and confidentiality were ensured, since the researcher did not have cumulative access to the participant’s identification and the collected data. The association with the data was made by an identification number.

### Data Analysis

To analyze the correlation between Global Management of Health Organizations dimensions was used Pearson correlation and T-Student test for group comparison (comparison between Time 1 and Time 2), IBM SPSS 22 was used.

To analyze the associations between the variables, a structural equation model (SEM) was used with the unweighted least squares method (ULS), JASP 0.14.1 program was used. Different adjustment indices Evaluated the fit of the model: Chi-square (*χ*2), Comparative Fit Index (CFI), Root Mean Square Error of Approximation (RMSEA) and Standardized Root Mean Squared Residual (SRMR). For CFI a value greater than 0.90 is considered appropriate and for RMSEA and SRMR the values near or below 0.08 and 0.05 were considered acceptable.

The intensity of the relationships of the variables was analyzed through standardized coefficients and their effect size (*η*2) and following the procedure of Peterson and Brown (2005). We considered a small effect size with values around 0.05, moderate effect for values from 0.06 to 0.11, and a large effect when the values were equal to or greater than 0.14 (Cohen 1988).

Finally, a configurational invariance analysis was realized according to the two groups: pre and post COVID-19. The adjustment indices of the models are presented considering an increase of 0.01 as an indicator of significant change in the models (Cheung and Rensvold 2002).

For the analysis of the open question the MAXQDA program was used, the participants' answers were organized into categories and subcategories based on the systemic model including the different political, organizational, professional, and patient systems Table 1.

**Table 1:** summary of literature review

Relevant information	References
COVID-19 pandemic that affected and killed millions of people worldwide	WHO 2022
Healthcare workers were at increased risk of infection	European Centre for Disease Prevention and Control 2020 WHO 2021
Healthcare workers were a high-risk group for stress and burnout	Hajure et al. 2021, Mohammadian 2021 Pfefferbaum 2020 Zandifar 2020
The COVID-19 pandemic, the large number of associated deaths and periods of confinement increased the prevalence of mental health problems, namely anxiety and depression	Organization for Economic Co-operation and Development (OECD), European Union 2020, Gaspar et al. 2021
Health professionals in Portugal were among the most infected people in a professional context	Campos 2021 Directorate-General for Administration and Public Employment 2021
Female health professionals were even more affected by the pandemic	WHO 2021 OECD 2020
The impact of the pandemic on the mental health of health professionals should be seen in a long-term perspective	Iskandarsyah et al. 2021 Muhammad et al. 2022
The impact of the pandemic on the mental health of health workers also has a negative impact on their patient care	Mohammadian 2021 Muhammad et al. 2022
The importance of implementing measures to promote health and prevent stress and burnout among health professionals is fundamental to mitigate and reduce the impact	WHO 2021 Rodríguez 2020
There are good practices associated with the management of psychosocial risks at work	Gaspar et al. 2021 Mohammadian 2021 Yáñez-Araque et al. 2021 Schmidt et al. 2022

## Results

In Table 2, it was sought to examine the correlations between the variables under study. A positive correlation was found between the variable Organizational Culture and the Psychosocial Environment related to Content and Leadership (*R* = 0.63; *p* < 0.001) and the variable Organizational Culture and Job Satisfaction (*R* = 0.66; *p* < 0.001).

**Table 2:** Correlations between dimensions

	1	2	3	4	5	6
1. Organizational Culture	1					
2. Quality of Life	0.29**	1				
3. Performance Management	0.18**	0.24**	1			
4. Psychosocial Environment related to Content and Leadership	0.63**	0.41**	0.27**	1		
5. Psychosocial Work Risks related to Mental Health	− 0.34**	− 0.59**	− 0.10*	− 0.46**	1	
6. Professional Job Satisfaction	0.66**	0.36**	0.28**	0.87**	− 0.47**	1

With regard to the variable Psychosocial Work Risks related to Mental Health, a negative correlation was found with Quality of Life (*R* = − 0.59; *p* < 0.001) and with Psychosocial Environment related to Content and Leadership (*R* = − 0.46; *p* < 0.001). Quality of Life has a positive correlation with Psychosocial Environment related to Content and Leadership (*R* = 0.41; *p* < 0.001), the positive correlation of Job Satisfaction with Psychosocial Environment related to Content and Leadership (R = 0.87; p < 0.001) and the negative correlation with Psychosocial Work Risks related to Mental Health (*R* = − 0.47; *p* < 0.001) are also highlighted.

### Comparative Results Between the Two Times (1 and 2) of Data Collection, 1 in November 2019 and 2 in November 2021

Regarding data collection, 37.0% of participants responded in the first Time (*n* = 296) and 63.0% responded in the second Time (*n* = 503).

Regarding the comparison pre and during pandemic COVID-19 (Table 3), it is found that the participants in the pre- COVID-19 era showed stronger organizational culture, higher quality of life, better psychosocial environment regarding content/leadership and higher job satisfaction when compared to the participants in the phase during pandemic COVID-19. On the other hand, participants in the second Time were found to have higher mental health Psychosocial Work Risks (PWR) when compared to participants in the pre- COVID-19 phase. Regarding Performance Management there are no statistically significant differences between the two occasions.

**Table 3:** Comparison pre and during pandemic COVID-19

	Occasion 1		Occasion 2		*t*/Sig
	M	DP	M	DP	
1.Organizational culture	3.13	0.62	3.10	0.73	2.25**
2.Quality of life	3.92	0.64	3.59	0.70	6.64***
3. Performance management	3.73	0.71	3.81	0.62	− 1.52(n.s.)
4. Psychosocial environment related to content and leadership	3.17	0.85	3.01	0.96	2.35**
5. Psychosocial work risks related to mental health	2.81	0.84	2.98	0.79	− 2.78**
6. Professional job satisfaction	2.56	0.50	2.43	0.53	3.45***

***p < 0.001; **p < 0.01; *p < 0.05

#### Mediation Model and Invariance across Dimensions

**Fig. 1:**
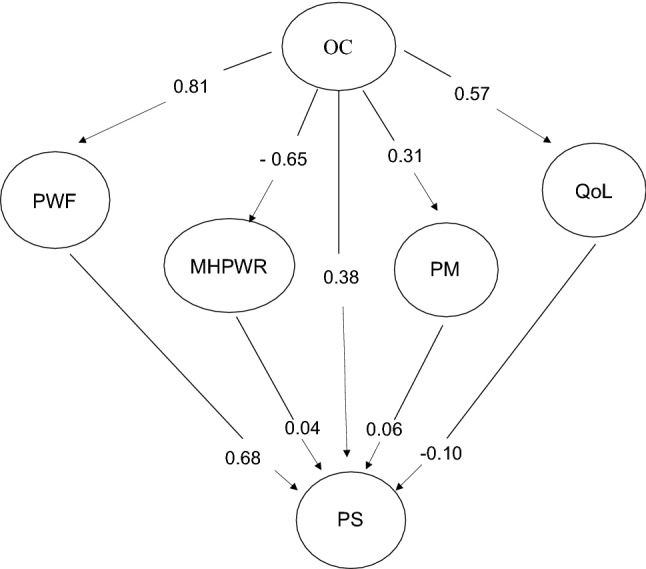
Representation of the standardized estimations of the path coefficients of the global model. *OC* Organizational Culture; *PWF* Psychosocial Work Factors; *MHPWR* Mental Health Psychosocial Work Risks; *PM* Performance Management; *QoL* Quality of Life; *PS* Professional Job satisfaction.

Table 3 showed excellent ft to the data (CFI = 0.981; RMSEA = 0.057; SRMR = 0.064; *χ*^*2*^*/df* = 3.57) and the Fig. 1 presents the standardized coefficient of the model estimating.

The model explained 90% of the variance of PS, 65.0% of the variance of PWF, 42.0% of MHPWR, 32.3% of QoL and of 0.09% of the variance of PM. The direct paths from OC to PS showed a positive and moderate intensity with large effect size *(β* = 0.38; *η*^2^ = 0.18). Regarding the direct effects from the OC on PWF and OC on QoL, results showed high factor loadings with large effect size. Direct associations between OC and PM were also positive and moderate with intermediate effect size. However, the relationship of OC on MHPWR was negative with a large effect size. On the other hand, there were no direct effects of MHPWR, PM and QoL on PS although, there was a positive and intense association of PWF on PS with a large effect size.

In addition to direct effects, a mediation effect of OC on PS was also observed through PWF (*β* = 0.55; *η*^*2*^ = 0.250). There were no indirect associations between OC and PS through MHPWR (*β* = 0.03; *η*^*2*^ = 0.00), nor through PM (*β* = 0.02; *η*^*2*^ = 0.00) or QoL (*β* = 0.06; *η*^*2*^ = 0.01).

Finally, configurational invariance analyses were realized with two groups: pre COVID-19 and post COVID-19 (Table 4) showing excellent adjustment and invariance with respect to the global model, since there was no increase in CFI greater than 0.01.

**Table 4:** Goodness of fit for the proposed factorial model and the configurational invariance analysis

	Global	Occasion 1 Vs occasion 2
*χ*^*2*^*/df*^*a*^	3.575	2.203
NNFI^b^	0.980	0.982
CFI^c^	0.981	0.982
IFI^d^	0.981	0.968
RMSEA^e^ (CI 95%) ^f^	0.057	0.055
SRMS^g^	0.064	0.071
CFI^h^	–	0.001

*χ*^*2*^*/df*^a^ chi-square/degree of freedom; ^b^*NNFI* non-normed fit index; ^c^*CFI* comparative fit index; ^d^ *IFI* incremental fit index; ^e^*RMSEA* root mean squared error;^f^*CI* confidence interval; ^g^*SRMR* standardized root mean squared residual; ^h^*CFI* increase in CFI

Open question “SUGGESTIONS FOR IMPROVING THE QUALITY OF HEALTH ORGANIZATIONS”.

The participants’ answers will be organized into categories and the subcategories identified will be indicated on the basis of the systemic and ecological model .

First the identification of the barriers, that were categorized in barriers related to management, related to professionals and related to patients. Then the overall impact of COVID-19 and then proposals for improvements in relation to the different systems.

Suggestions for improving the quality of health organizations are indicated at the political level, management level, related to professionals, patients, information and communication systems and innovation Tables 5, 6.

**Table 5:** Barriers related to quality of health organizations

	Barriers
Management	Regarding the main barriers/weaknesses to increasing the quality and effectiveness of NHS health organizations that are linked to management the experts revealed high agreement regarding: -Lack of strategic planning -Lack of leadership and failures in internal communication -Very bureaucratic and disperse reporting system -Lack of autonomy from central power -Underfinancing of the health organization
Professionals	In relation to the professionals, the specialists revealed a high level of agreement regarding: -Lack of incentives associated with performance -Demotivation -Work overload
Patients	In relation to patients, the specialists revealed a high level of agreement regarding: -Waiting times -Weaknesses at the level of access conditions -Poor patient literacy and empowerment
COVID-19	Many of the participants agree that the pandemic caused by COVID-19 brought about substantial changes at the Organizational level (organizational culture and autonomy) and factors related to health professionals (psychosocial work factors; quality of life and perception of performance) and their influence at the outcomes level (professionals’ satisfaction, patients’ satisfaction and economic-financial factors) in health organizations

**Table 6:** Suggestions for improving the quality of health organizations

Policy level	Regarding priority improvements at the political level the professionals agree on the need for: -Governance–transparency and regulation -Long-term strategic planning -Adequate financing of NHS health organizations -Greater management autonomy in relation to central and political power -Dissemination and extension of good practices (centers of reference, etc.) -Increase in the financial autonomy of NHS health organizations -Debureaucratization (hiring HR, procurement of goods and services, etc.)
Management level	In relation to improvements at management level 90% or more of the experts agree with the need for: -Long-term strategic planning taking into consideration the optimization of services -Improved internal communication between different hierarchical levels -Regular audits of clinical management and practice -Improve physical conditions of health care organizations -Reduce waste-resources and materials
Professionals level	In terms of the improvements related to professionals the participants agree with the need to: -Review the remuneration of professionals, institute financial incentives (bonuses) according to performance appraisals -Greater autonomy, involvement and accountability -Better working conditions for health professionals (overall) -Promote the continuous training of professionals -Possibility and articulation with other activities (research, etc.) -Non-financial rewards
Patient level	Regarding the priority improvements related to patients the professionals agree with the need to: -Put the patient at the center. Promote triangulation of processes: they should be patient-centered, professional-led and manager-led in conjunction with patients -Assess patient satisfaction and set up an effective system to respond to complaints and compliments -Increase the health literacy of the population
Information and communication systems level	Regarding the improvements at the level of information and communication systems the experts agree with the need for: -Robust, agile and integrated clinical information system -A technologically advanced communication and information system that allows for processes and procedures to be homogeneous
Level of innovation	Linked to innovation the participants agree with the need for: -Systematic connection to the community/network -Home care -Home hospitalization -Valorization of extra-health care activities (research, teaching, training, etc.)

## Discussion

The results obtained are key to achieve the proposed objective, namely, to understand and characterize the impact of COVID-19 at the hospital management level, psychosocial environment, and satisfaction of Health Professionals through the case study of a Portuguese public hospital before and almost two years after COVID-19.

The authors found that the dimensions of the overall management of the health organizations under study are statistically significantly correlated, namely the relationship between the Organizational Culture and the Psychosocial Environment related to the work content and leadership, the Psychosocial Risks of Work related to the wellbeing and mental health, the Quality of Life of Professionals, the Professionals’ Satisfaction with the Organizational Culture and the Professionals’ Satisfaction and Psychosocial Environment related to the work content and leadership.

Recent literature confirms that the organizational culture related to values, goals, relational climate, success indicators, type of leadership, etc. has an influence especially on the psychosocial work environment related to leadership and work, namely the perception of justice, respect, recognition and career development opportunities. However, studies have shown that if leadership are strong, consistent, and engaged health professionals, even submitted to high pressure and stress at work, will not hesitate to be involved in projects and give their best work because they feel engaged and committed (Yáñez-Araque et al. 2021).

The organizational culture also strongly impacts on the satisfaction of professionals with the support, involvement, empowerment, opportunity, and communication within the organization. A culture that prioritizes wellbeing at work, a positive relationship and communication with the leadership and with the work performed, a real involvement and fairness with the professional are fundamental for a better functioning of the whole organization and its human resources (Berghofer et al. 2020; Braithwaite et al. 2020; Burton 2010; Jackson et al. 2020; OECD 2020).

Our results regarding the comparison pre and during pandemic COVID-19 reveals that the participants with COVID-19 showed less positive organizational culture, lower quality of life, worst psychosocial environment regarding content/leadership, more psychosocial risks related to stress and burnout and low job satisfaction when compared to the participants in the phase during pandemic COVID-19.

This is similar to what is pointed by EU-OSHA study (European Agency for Safety and Health at Work 2021) that health organizations are faced with several challenges in relation to the mission and outcomes they set out to achieve, COVID-19 has compounded and complexified these challenges. Health and wellbeing, as well as staff and patient satisfaction, will influence outcomes (European Agency for Safety and Health at Work 2021; OECD 2018, 2020a, 2020b).

Psychosocial risk factors can be individual factors, organizational factors and the interaction between them, the level of exposure to the risks and the concrete impact it has on the general health of the professional (Singh and Conroy 2017). Our study identified some individual, psychological, relational, and organizational risks. Literature also describes psychosocial risks at work, such as coping style, personality and cognition characteristics and sociodemographic characteristics, such as gender, age, marital status and clinical history at the level of physical and mental health (Gaspar et al. 2021; OECD 2020; Yanez-Araque et al. 2021; WHO 2021; European Agency for Safety and Health at Work 2021).

Direção-Geral da Saúde (2015a, b) describe the main factors that should be considered when assessing psychosocial risks at work: the culture of the organization and leadership relationships, work content, workload, working hours, level of control and autonomy, interpersonal relationships at work, possibilities for career development, work-family relationship, and work environment and equipment. The impact of psychosocial risks on the health and performance of professionals can have chronic and long-term consequences (OECD 2018, 2020a, 2020b), at the level of their physical health (Iskandarsyah 2021), mental health and chronic stress (Giménez-Espert et al. 2020) and work absenteeism (van der Plaat et al. 2021; WHO; 2021). Health professionals are among those who suffer most from psychological stress and are also among those with the highest risk of burnout and many of these symptoms are long-term symptoms, including chronic stress, increased incidence of depression and anxiety, increased consumption of problematic substances and behaviors, and increased absenteeism (Gaspar et al. 2021).

In this particular COVID-19 situation all this occupational risk become overwhelmed because health care professional, as front line of care, need to deal with the suffering of patients and their families, they need to managed all the emotional stress regarding the unknown of this new disease, the afraid of being infected and infect their families and, also, afraid of the comorbidities and death (Mohammadian et al. 2021; Pfefferbaum and North 2020; Zandifar et al. 2020).

Recent studies evidence that increased fear levels relating to COVID-19 have a relationship with lower levels of job satisfaction, higher levels of job turnover (Abd-Ellatif et al. 2021) and high rates of work-related illness, rates of absenteeism and less productivity (WHO 2021).

The model under study highlights that professionals' satisfaction decreased with COVID-19 and that it is influenced by the other dimensions of healthcare organizations. A more positive and well-defined organizational culture and values are related to higher professional satisfaction. In turn, the organizational culture has a strong impact on the psychosocial environment, the psychosocial risks of work related to stress and burnout, and the professionals’ quality of life.

The COVID-19 pandemic has negatively impacted job satisfaction among healthcare workers (Alrawashdeh et al. 2021; Amer 2021; Mendonça-Galaio et al. 2020). Inadequate preparedness, stress, and burnout are significant contributing factors. The pandemic crisis obligates all the health care professionals to be prepared for a crisis without all the information detailed and known, without sufficient access to personal protective equipment (Alrawashdeh et al. 2021.)

During the COVID-19 pandemic, healthcare professionals might have experienced mental health problems and work-related stress, which can lead to less satisfaction at work and decreased health and quality of life in the long period (Gaspar et al. 2020; Iskandarsyah et al. 2021).

Our model reinforces the interrelationship and influence between the variables allows for the identification and understanding of organizational, professional and outcome factors and their relationship that are influenced by organizational culture, professionals ‘quality of life, psychosocial risks and outcomes (Anderson 2016; Berghofer et al. 2020; Ivey et al. 2018; Gaspar 2020; Okunade et al. 2017; Schmidt et al. 2022).

With regard to the suggestions for improvement made by the participants, the following are highlighted:

To improve the quality and results of health organizations, there is a fundamental need for transparency and regulation in governance, autonomous management that is independent from political power, long-term strategies, multi-annual plans and budgets, hospital managers with management training, management autonomy and accountability, systematic evaluation of processes and results, improved hierarchical communication. Valorisation and rationalization of existing resources. Financing must be patient-centered and in accordance with the organization's performance. It is fundamental to have articulation between the different levels of service provision and greater investment in prevention, systematic connection with the community/network and home care. The fundamental importance of having a robust, agile clinical Information and Communication System. In terms of e-health, there must be greater investment in the use of telemedicine and other specialties as a way of expanding responses in health (Anderson 2016; Berghofer, et al 2020).

Regarding professionals, the need to review professional remuneration, to institute financial incentives (prizes) according to performance evaluations, to promote greater autonomy, involvement and accountability of professionals, and to improve the level of psychosocial risks at work, namely in terms of stress and burnout, must be stressed.

At the level of health systems evaluation and monitoring, it is proposed to set up a multidisciplinary working group, which includes professionals from various health institutions, with the mission of presenting practical, concrete and objective proposals.

Define few goals and to be achieved in well-timed times with the use of realistic resources. Need to promote an annual event for discussion among hospital directors representing the NHS, the order of physicians, patient associations and other institutions deemed relevant. Finally, is important share/elaborate a catalog of the best practices of the National Health System (Alrawashdeh, H.M. et al. 2021; Direção Geral da Saúde 2022; Muhammad et al. 2022).

Given the already strained healthcare system and low morale among healthcare workers, efforts are needed to increase preparedness, quality of life, better stress management and burnout, and improve professional job satisfaction and involvement especially during the pandemic. The protection of the mental health of workers should be integrated into workplace occupational safety and health management systems (OSH-MS), emergency preparedness and response plans and return to work plans developed to respond to the COVID-19 crisis. The intervention should cover all the different dimensions and risks related to work environment and organization, including psychosocial factors. As have been showed significant physical and psychological burden was associated with the COVID-19 pandemic. Important efforts should be implemented to protect health care professional wellbeing, enhancing their working conditions, and raising awareness about burnout. Proper utilization of financial and human resources are crucial for the sustainability of the health care system and the health care workforce, especially in crises (Yanez-Araque et al 2021; Van Gool et al 2022; WHO 2021).

According to guidelines prevention and control procedures should: (a) be adapted to the challenges and risks encountered by the organization; (b) be reviewed and adapted, if necessary, on a regular and continuous basis; (c) articulated with national laws and regulations, and reflect good practice; and (d) take in account “state of art” actual knowledge, including information or reports from governmental and non-governmental organizations, universities, etc. (International Labour Organization 2020).

The OECD (2017) and Clarkson et al. (2018) argue that the quality assessment of health organizations, from a systemic perspective, should take into account several key concepts, such as leadership, strategy, plans, patients, society, information and knowledge, people, processes, and outcomes.

To improve overall health, the health system needs a tripartite strategy involving education and training, practice and research (Day-Duro et al. 2020). A strong focus on management, professional satisfaction and wellbeing, commitment to healthy workplaces as fundamental elements for global health, can open new and sustainable paths to improve health systems performance (Bradley et al. 2015; Braithwaite et al. 2020; Gaspar et al. 2021).

## Conclusions

The comparative analysis between the two study moments in time (before and 2y after the beginning of pandemic) shows that there is, in general, a worsening in the professionals' perceptions. At time 2, professionals had lower mean scores regarding the perception of organizational culture, revealed a less positive perception of quality of life, a psychosocial work environment related to work content and leadership with higher risk, identified higher psychosocial work risks related to mental health, and reported lower satisfaction with supervisors and the work performed.

Adherence to the questionnaire was higher at occasion 2 which may be a weakness of the study, however it may also reflect that after two years of facing the pandemic, professionals were exposed to high levels of stress and higher psychosocial risks at work which may have been reflected in their willingness and interest to participate in the study and to be involved in strategies to improve the health and quality of health organizations.

The in-depth study of the professionals’ satisfaction reveals that it is influenced by all the dimensions under study, but the key role of the psychosocial environment related to the work content and leadership relationship, as well as the organizational culture, should be highlighted.

This knowledge facilitates labeling and prioritizing the promotion of wellbeing and satisfaction of professionals and overall wellbeing of the health organization, namely intervention in leadership relationships and psychosocial risks at work related to the physical and psychological demands of work, stress management and a more active and involved role for the professional.

The product is a comprehensive diagnostic model of the factors influencing the results in health organizations that allows for a greater knowledge of the systems and the relationships between systems and that can support decision-making, planning and implementation of improvements in health organizations. For the regular assessment and monitoring of health organizations, an integrated assessment model and method was studied and proposed to evaluate the impact of the implementation of improvement measures and consequently support an evidence-based governance process.

Some implications for practice from the results, recommendations and products of this study are highlighted: supports the identification of the greatest needs and presents concrete consensual proposals for improving the quality and services provided by the National Health Service (NHS); at the organizational level (administration and management), the importance and impact of organizational culture on processes and results was demonstrated; At the professional level, the relevance of involving all professionals was mirrored, groups with specific needs and levels of need were identified, and the relationship between QoL and psychosocial risks of work with other processes and outcomes of health care organizations was confirmed; at the patient level, there was a general satisfaction with the HS, especially with the organization under study.

The main recommendations for managers of public health organizations are the following:

- Evaluate and monitor the quality and performance of health organizations from a systemic and integrative perspective (integrating inputs, processes and outcomes) and on a regular basis;
- The results of the evaluations should be returned to and discussed with all stakeholders (or representatives), specific and realistic objectives and goals should be established, and consequently the necessary and pre-established changes planned, implemented and evaluated;
- Establish and communicate transparently and consistently the organizational culture;
- Promote better communication and transparency between different hierarchical levels and different professional groups;
- Promote Healthy Workplaces by improving psychosocial working conditions, promoting physical, social and mental health of professionals, for example by giving them more availability for extra-care activities such as the acquisition of management, research and training qualifications;
- Human resources strategy that rewards performance, incentives according to the evaluation of the professionals' performance;
- Active involvement of professionals in their performance management;
- Recognition of professionals for their experience and training, professional valorisation by objectives and autonomy in hiring in the organizations;
- Promote at the professional level an improvement in communication and teamwork, and training in context, for an effective standardization of procedures.

The results indicate that the pandemic COVID-19 had an impact on the health and mental health of the population, and health professionals were one group that felt this negative effect most strongly. The impact of COVID-19, coupled with all the other social and economic changes, can have medium to long-term effects. Health care organizations and professionals, and society as a whole, were generally very resilient during and in the fight against the pandemic. The mental health of health care workers has an influence on the delivery of health care and consequently on the health of patients. Mental health was a major factor in the management of the pandemic, it is now essential, on the one hand, to promote interventions and policies directly related to health promotion of health workers and promotion of healthy work environments to cope with COVID-19 and on the other hand anticipate social, economic and health crises that are occurring or may arise in the future. It is recommended to promote system resilience and sustainability by promoting and strengthening mental health and wellness in health care organizations.

## Data Availability

The datasets generated and analyzed during the current study are available from the corresponding author on reasonable request.
